# The Identification of Novel Diagnostic Marker Genes for the Detection of Beer Spoiling *Pediococcus damnosus* Strains Using the BlAst Diagnostic Gene findEr

**DOI:** 10.1371/journal.pone.0152747

**Published:** 2016-03-30

**Authors:** Jürgen Behr, Andreas J. Geissler, Jonas Schmid, Anja Zehe, Rudi F. Vogel

**Affiliations:** Lehrstuhl für Technische Mikrobiologie, Technische Universität München, Freising, Germany; University of Illinois at Chicago, UNITED STATES

## Abstract

As the number of bacterial genomes increases dramatically, the demand for easy to use tools with transparent functionality and comprehensible output for applied comparative genomics grows as well. We present BlAst Diagnostic Gene findEr (BADGE), a tool for the rapid prediction of diagnostic marker genes (DMGs) for the differentiation of bacterial groups (e.g. pathogenic / nonpathogenic). DMG identification settings can be modified easily and installing and running BADGE does not require specific bioinformatics skills. During the BADGE run the user is informed step by step about the DMG finding process, thus making it easy to evaluate the impact of chosen settings and options. On the basis of an example with relevance for beer brewing, being one of the oldest biotechnological processes known, we show a straightforward procedure, from phenotyping, genome sequencing, assembly and annotation, up to a discriminant marker gene PCR assay, making comparative genomics a means to an end. The value and the functionality of BADGE were thoroughly examined, resulting in the successful identification and validation of an outstanding novel DMG (f*abZ*) for the discrimination of harmless and harmful contaminations of *Pediococcus damnosus*, which can be applied for spoilage risk determination in breweries. Concomitantly, we present and compare five complete *P*. *damnosus* genomes sequenced in this study, finding that the ability to produce the unwanted, spoilage associated off-flavor diacetyl is a plasmid encoded trait in this important beer spoiling species.

## Introduction

### Background

In February 2016 the Genomes OnLine Database counted 52,366 prokaryotic sequencing projects, representing more than 65% of all available sequencing projects [1]. The number of next generation sequencing (NGS) projects will increase in the future as sequencing technologies like single molecule real time sequencing (SMRT) produce high quality data at decreasing costs, mostly resulting in complete genomes [2–4]. Consequently, comparative genomics studies will gain importance in order to identify strain or group specific genetic traits. Thereby the approach of comparative genomics is and will be relevant in any section of microbiology, from medical microbiology to food microbiology and others [5–7]. It is unlikely that all users utilizing comparative genomics are and will be bioinformatics scientists, especially if we consider NGS and comparative genomics as a means to an end tool in the future, like Sanger sequencing of small DNA molecules is currently. Therefore the demand for easy to use tools, at best application-oriented, easy to install and handle, will increase. We present BlAst Diagnostic Gene findEr (BADGE), a powerful yet still user-friendly tool for the rapid prediction of diagnostic marker genes (DMGs), valuable for diagnostics, large scale strain screening or selection for desirable traits.

While the term of DMGs is mostly related to medical diagnostics, we could generally consider DMGs as genes correlated with a specific trait or phenotype of a microorganism. This can be the ability to produce exopolysaccharides and a capsule, to grow at a high salt content, to invade a certain host or to colonize a specific habitat, designating an ecotype. These markers should not be confused with popular molecular markers like the 16S ribosomal RNA (rRNA), *rpoB* or *recA* genes. These are useful for taxonomic classification due to their conserved and polymorphismic character, mostly being housekeeping genes present in all bacterial species [8]. While they can be used to differentiate between species or genera, they do not necessarily help to distinguish different phenotypes. The knowledge of DMGs with a good correlation to a certain phenotype is a very powerful tool. In medical diagnostics, DMGs are generally virulence genes and can help to differentiate between pathotypes, as done for enteric pathogenic *Escherichia coli* [9, 10]. In food technology they can help to detect undesired spoilage organisms, as shown in the case of beer spoiling bacteria [11, 12]. From the microbe´s perspective, DMGs represent essential features which enable bacteria to occupy a specific ecological niche.

Beer represents such a specific ecological niche, characterized by various antibacterial hurdles that prevent the growth of most bacteria. [13]. During the fermentation of hopped, mainly barley based wort by yeast, ethanol and CO_2_ are produced and the pH is lowered. This activates the antimicrobial properties of hop bitter acids (manly α-acids isomerized during wort boiling to iso-α-acids), resulting in a microbiologically very stable beverage [14, 15]. Iso-α-acids have two modes of action. They act as protonophores, dissipating the transmembrane gradient, and they induce transmembrane redox reactions, which cause oxidative stress [15–17]. Some lactic acid bacteria (LAB) species are still capable of growing in beer, and this ability has been correlated with the presence of hop resistance genes in different studies. Prominent hop resistance associated DMGs, such as *horA*, *horC* and *hitA*, were originally identified for *Lactobacillus brevis*, while their relevance was also shown or suggested for other beer spoiling LAB. Hop resistance (*hor*) gene A and C were predicted to act as hop efflux transporters, while the hop inducible transporter (*hit*) A is considered to play an important role in maintaining the intracellular Mn^2+^ content [18–20].

Between 1980 and 2002 *Pediococcus damnosus* caused 12% of all beer spoilage incidents in Europe, making this species the second most important spoilage organism after *L*. *brevis*. Spoilage by *P*. *damnosus* leads to sediment formation, acidification as well as off-flavors (diacetyl) in beer. This results in an inedible product and consequently economic damage. Classical tests for beer spoilage ability are very time-consuming, as they are mostly cultivation based [13]. Therefore fast and reliable molecular detection methods are desired in order to detect threatened batches as early as possible. *P*. *damnosus* is characterized by a strain specific beer spoilage potential. As strains with different spoilage potential might be met in the brewery environment, it is impossible for brewery microbiologist to evaluate the current risk of beer spoilage based on species identification. In such cases, DMGs for the identification of the strain specific ability to grow in beer and to occupy this specific ecological niche are required.

### Current approaches for the prediction of DMGs

The following tools are generally capable of predicting DMGs: CMG biotools, EDGAR, Gegenees and Panseq [21–24]. While being very useful tools, none of them focuses on the applied prediction of DMGs, as either laborious post-processing is necessary or only scarce possibilities for changing settings are given.

The web-based version of Panseq is platform independent and a standalone version for Linux is available, relying on multiple dependencies, e.g. Perl and Bioperl. Panseq identifies any genomic regions present in any strain of a target group A that are not present in any strain of a background group B on DNA level [21]. Iterative processing is necessary to retrieve regions present in every strain of group A and in order to predict potential DMGs encoded on the found regions.

CMG biotools comes as a modified version of an Ubuntu operating system (OS). It is based on different programming languages and includes useful tools for comparative genomics, e.g. tools to calculate pan- and core-genomes on protein level. Iterative calculation of intersections, unions and complementary gene sets is possible. This enables the user to extract group specific proteins in a variable manner, e.g. present in all or just 75% of group A genomes, but not in any genome of group B [22]. A consecutive processing and filtering is necessary in order to obtain DNA sequences of potential DMGs. This is needed, since highly homologous protein sequences within the target group do not necessarily correspond to highly homologous nucleotide sequences (degeneracy of the genetic code). Consequently, the suitability of a DMG for a targeted identification by PCR or any other DNA-based method may be restricted due to the low DNA sequence similarity of a given DMG within the target group.

EDGAR is “a software framework for the comparative analysis of prokaryotic genomes” [23], it is web-based and therefore platform-independent. Like CMG biotools, it includes useful tools for comparative genomics and is written in various programming languages. The user is able to predict potential DMGs based on precomputed comparisons [23]. There is no standalone version, private projects are calculated only on request and subsequently settings cannot be changed ‘spontaneously’.

Gegenees is user friendly, written in JAVA and comes as a platform independent stand-alone tool with a graphical user interface. Fragmented alignments of whole genomes are created and can be used to extract regions that are characteristic for one specific target group in a variable manner. Three different settings with respect to DMG quality can be chosen and biomarker scores are assigned, enabling the user to sort the found DMGs according to their quality [24]. However, post-processing is necessary for workup and selection of DMGs for any further procedure. In addition, there is no possibility to change the settings of blast, which is used as the central genome comparison tool by Gegenees.

### Motivation

**Fig 1:**
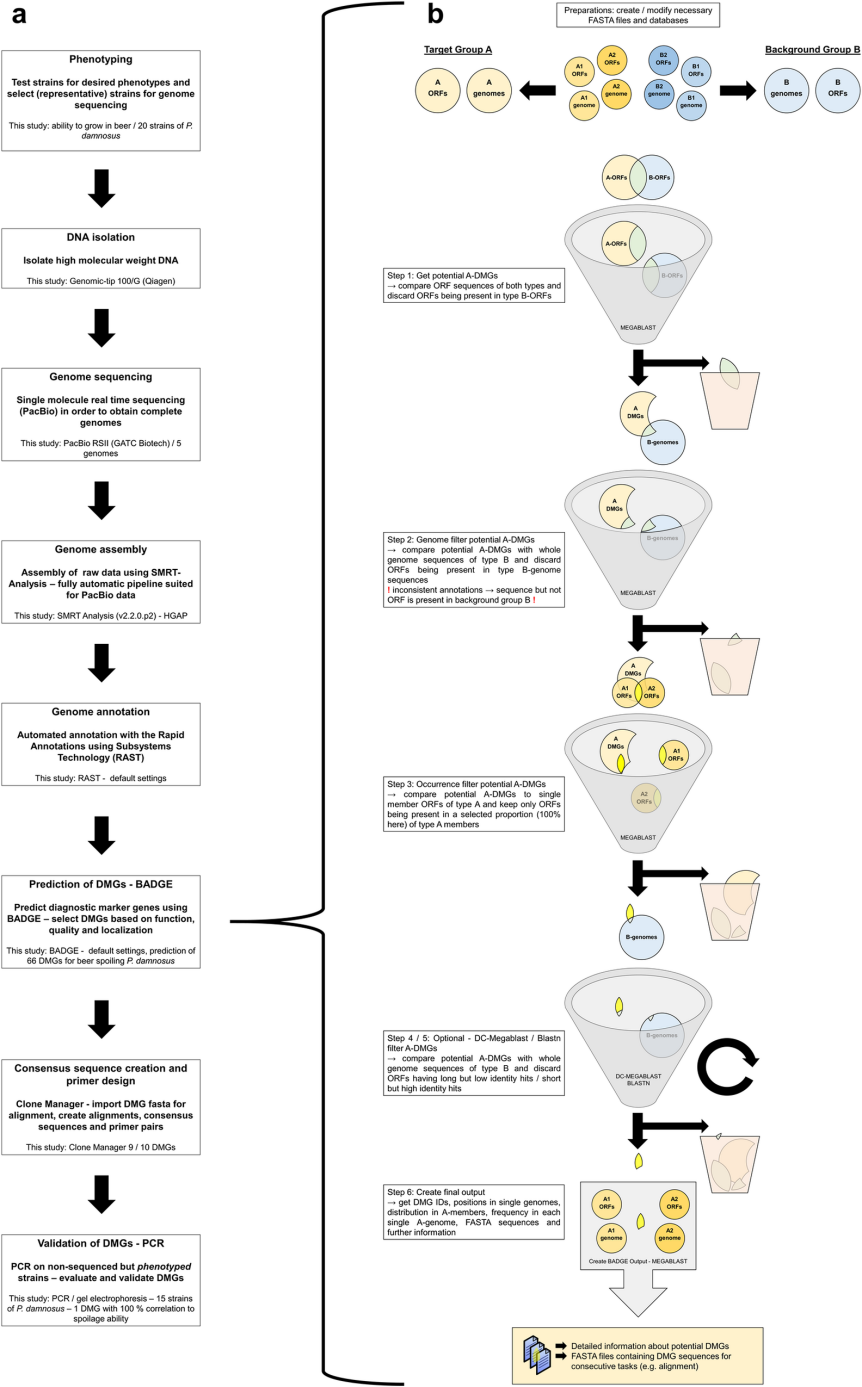
The use-oriented prediction of diagnostic marker genes using BADGE. (a) Workflow illustrating the structure of a comparative genomics project with the aim to identify and validate diagnostic marker genes (DMGs). Each box corresponds to a single step within the straightforward procedure. (b) The prediction of group A specific DMGs is shown using a fictive example with two members of group A and two members of group B. A description of all steps can be found in the corresponding boxes. Genome = here: sequences of chromosome and all extrachromosomal elements (contigs); ORFs = open reading frames, here: sequences of all ORFs; DMG = diagnostic marker gene.

As described above, some approaches and tools for the prediction of DMGs are available. However, these tools partially lack ease of use, require the installation of additional software packages or lack flexibility. This makes them difficult to install and handle for users without programming or IT background. In addition they do not focus on an application-oriented prediction of DMGs, relying on subsequent post-processing of data. With BADGE we provide users with a fast and applied tool for the prediction of DMGs. BADGE is based on the sh-compatible command language BASH (Bourne-again shell). BADGE requires as few commands as necessary and is simple to install on unix-based platforms, while being easy to use and highly flexible. Within a straightforward procedure (see Fig 1) we used BADGE as a central tool for the prediction of DMGs for a strain specific trait, starting with phenotyping up to the point of validation of the predicted DMGs using PCR. We sequenced five genomes of the important beer-spoiling species *P*. *damnosus*, comprising strains with varying beer spoilage ability. After assembly to complete genomes and consequent annotation, we used BADGE to identify new DMGs, allowing the differentiation of *P*. *damnosus* strains according to their ability to grow in and spoil beer.

## Methods

### Bacterial strains, media and culture conditions

Table 1 lists all 20 strains as well as their origin. In order to obtain a representative batch of strains, brewery isolates were obtained from 16 different sources, varying in isolation date and geographical location. Strains from the winery environment originate from two different sources. If not stated differently, cells were cultured at 25°C using a modified MRS medium (mMRS_1_) with a pH of 6.2 as previously described [25]. Upon receipt and before storage at -80°C in 40 vol. % glycerol, all isolates were propagated four times on mMRS_1_ using single colony transfer. This was done in order to get strains into a comparable physiological state and to separate possibly mixed cultures. Species membership of all strains was confirmed using Matrix-Assisted-Laser-Desorption-Ionization-Time-Of-Flight Mass Spectrometry, which is a powerful tool for the identification of brewery environment bacteria [26].

**Table 1:** Strains, alternative identifiers (DSMZ), source of isolation and spoilage potential as determined by beer spoilage test. TMW = Technische Mikrobiologie Weihenstephan. OD_590_ and pH values determined after 60 days of incubation can be found in S1 Table. Growth in test beers is indicated by + and -. IBU = international bitterness units (measure for hop content). Spoilage-groups: strong beer spoilage potential (SB)—growth in pilsner beer, middle potential (MB)—growth in lager beer, weak potential (WB)—growth in wheat beer, no potential (NB)—no growth in test beers.

Strain / Alternative identifier	Source	Growth in wheat beer 1, pH 4.4, 14 IBU, 5.5 vol. % alcohol	Growth in lager beer 1, pH 4.3, 18 IBU, 5.1 vol. % alcohol	Growth in lager beer 1, pH 4.4, 29 IBU, 5.1 vol. % alcohol	Spoilage potential
**TMW 2.4 / DSM 20331^T^**	lager beer yeast	+	+	-	MB
**TMW 2.125**	unknown	-	-	-	NB
**TMW 2.1532^S^^X^**	bottled beer	-	-	-	NB
**TMW 2.1533^S^^X^**	brewery environment	+	+	+	SB
**TMW 2.1534^S^^X^**	brewing yeast sample	-	-	-	NB
**TMW 2.1535^S^^X^**	pilsner beer	+	+	+	SB
**TMW 2.1536^S^^X^**	winery environment	-	-	-	NB
**TMW 2.1546**	brewery environment	-	-	-	NB
**TMW 2.1547**	pilsner beer—unfiltered	-	-	-	NB
**TMW 2.1548**	brewery environment	+	+	-	MB
**TMW 2.1549**	winery environment	-	-	-	NB
**TMW 2.1635**	*Kellerbier*	-	-	-	NB
**TMW 2.1636**	*Kellerbier*	+	+	+	SB
**TMW 2.1637**	brewing environment	+	+	+	SB
**TMW 2.1638**	brewery fermentation tank	-	-	-	NB
**TMW 2.1639**	brewery environment	-	-	-	NB
**TMW 2.1640**	wheat beer	-	-	-	NB
**TMW 2.1641**	brewery environment	+	+	+	SB
**TMW 2.1642**	pilsner beer—unfiltered	-	-	-	NB
**TMW 2.1643**	winery environment	-	-	-	NB

^T^ = type strain

^S^ = sequenced strain

^X^ = beer spoilage potential determined in a previous study [27]

mMRS_1_: 10 g/l peptone, 5 g/l yeast extract, 5 g/l meat extract, 4 g/l K_2_HPO_4_, 2.6 g/l KH_2_PO_4_, 3 g/l NH_4_Cl, 1 g/l Tween80, 0.5 g/l cysteine-HCl, 10 g/l maltose, 5 g/l glucose, 5 g/l fructose, 0.2 g/l MgSO_4_∙7H_2_0, 0.038 g/l MnSO_4_∙H_2_O

### Evaluation of beer spoilage potential

Beer spoilage potential was tested with a common beer spoilage test [27–29]. All beers were vacuum degassed and sterile filtered (0.2 μm, Thermo Fisher Scientific). The test was performed with three biological replicates at 25°C incubation temperature. Lager beer 1 with 18 International Bitterness Units (IBU) was adjusted to pH 5.0 (= lager_pH5.0_) with NaOH and inoculated with 2% of an mMRS_1_ pre-culture. 10 ml degassed test beers, wheat beer 1 with 14 IBU, lager beer 1 with 18 IBU (= lager_pH4.3_) and pilsner beer 1 with 29 IBU were inoculated with 5 x 10^3^ cells/ml and incubated for 60 days. Cells for inoculation were grown in lager_pH5.0_ for pre-adaption. Depending on visible growth in the test beers, strains were classified into four spoilage-groups: strong beer spoilage potential (SB)—growth in pilsner beer, middle potential (MB)—growth in lager beer, weak potential (WB)—growth in wheat beer, no potential (NB)—no growth in test beers. Strains with the ability to grow in at least lager_pH4.3_, were defined as beer spoiling strains (beer spoilage ability). After 60 days the pH was determined as well as the optical density at 590 nm. Both parameters were obtained to assist the visual assessment of growth, as acidification and turbidity are measures for lactic acid bacteria growing in beer. Triplicate samples of all beers without inoculation served as controls.

In order to test the effect of an additional fatty acid source on the spoilage ability of *P*. *damnosus*, lager beer 1 was supplemented with a Tween mixture (Tween 80, 60, 20, 1 g/l each). Inoculation, incubation and evaluation were done as described for the beer spoilage test. Five strains (TMW 2.1532, TMW 2.1535, TMW 2.125, TMW 2.1639, and TMW 2.1643) were tested for growth in lager beer 1 with Tween, while they were also inoculated into lager beer 1 without Tween as control.

For a selection of four strains (TMW 2.1532, TMW 2.1533, TMW2.1535, TMW 2.1536) we tested the transferability of the classification into spoilage potential groups obtained for the beers of brewery 1. Therefore we conducted a beer spoilage test, using the respective test beers obtained from three alternative breweries. Table 2 summarizes the most important parameters of the utilized beers, including those obtained from brewery 1, which were used for the classification of all strains. The resulting spoilage potential groups were then compared to each other for consistency.

**Table 2:** Beers used for beer spoilage tests. ID-Numbers (1 to 4) refer to different breweries. IBU = international bitterness units (measure for hop content).

**ID**	**Fermentation type**	**Special**	**Gravity (wt. %)**	**Alcohol (vol. %)**	**pH**	**IBU**
**Wheat beer 1**	top fermented	Kristall	12.5	5.5	4.4	14
**Wheat beer 2**	top fermented		12.7	5.3	4.2	13
**Wheat beer 3**	top fermented	organic	12.44	5.4	4.4	11
**Wheat beer 4**	top fermented		12.4	5.4	4.3	12
**Lager beer 1**	bottom fermented		11.5	5.1	4.3	18
**Lager beer 2**	bottom fermented		11.7	4.9	4.3	22
**Lager beer 3**	bottom fermented	organic	12.02	4.8	4.5	21
**Lager beer 4**	bottom fermented		11.58	4.9	4.6	17
**Pilsner beer 1**	bottom fermented		11.5	5.1	4.4	29
**Pilsner beer 2**	bottom fermented		11.9	4.9	4.5	33
**Pilsner beer 3**	bottom fermented	organic	11.15	4.7	4.5	27
**Pilsner beer 4**	bottom fermented		11.27	4.9	4.4	28

### DNA isolation, genome sequencing, assembly, annotation and analysis

High molecular weight DNA was isolated using the Genomic-tip 100/G kit (Qiagen). Quality and quantity was checked by NanoDrop (Thermo Fisher Scientific) and agarose gel electrophoresis. Single molecule real time sequencing (Pacific Biosciences (PacBio) RSII) of genomic DNA was carried out by GATC Biotech (Konstanz, Germany). An insert size of 8–12 kb was selected for library creation and an output of more than 200 Mb raw data, resulting from 1–2 SMRT cells (1×120 min movies) was generated using P4-C2 chemistry. Assembly was done with SMRT Analysis v2.2.0.p2, using the hierarchical genome assembly process (HGAP) [3] and manual curation as described by PacBio (https://github.com/PacificBiosciences/Bioinformatics-Training/wiki/Finishing-Bacterial-Genomes). All chromosomes and all plasmids, with one exception, were closed before annotation was carried out with the Rapid Annotations using Subsystems Technology (RAST) using default settings [30, 31]. Annotation was completed using RAST2BADGE, an included BADGE tool, creating modified fasta files from RAST fna and gff files. Annotated genomes were submitted to Genbank. Accession numbers are found within S2 Table. General genomic features, such as GC content or coding density, were calculated using BASH tools, such as “awk” or “sed”. Core genome and pan genome calculation was performed using CMG biotools applying default settings [22].

### Prediction of DMGs using BADGE

BADGE was installed on a personal computer (AMD Athlon™ X2 250 processor 3.00 GHz, 4.00 GB RAM) with Microsoft Windows 8 64 bit as host system, using VirtualBox 4.3.20 (www.virtualbox.org) and Ubuntu 14 (14.04.2 LTS, 64 bit) as guest system. The guest system was supplied with 1536 MB memory. BADGE was executed on the guest system using default settings (S3 Table). BADGE was also successfully tested with CentOS Linux release 7.1.1503, Ubuntu 12.04.5 LTS (32 bit), Debian GNU/Linux 8, openSUSE 13.2, Fedora release 21, Linux Mint 17.1 and OS X Yosemite 10.10.3. A detailed guide for the installation and execution of BADGE can be found in the BADGE manual. Associated fasta files containing the genome sequences and the ORFs were named identically and placed into the *genomes* and the *orfs* folders. Within these folders they were subsequently moved into two folders named *strong_spoiler* (TMW 2.1533, TMW 2.1535) and *non_spoiler* (TMW 2.1532, TMW 2.1534, TMW 2.1536), respectively, according to their spoilage potential. BADGE was started from the command line and output was generated within 3 min. DMGs were selected based on functional and spatial clustering using the final tabular output file (S4 Table). For a reliable prediction of their function, DMGs were further analyzed using NCBI blast [32, 33] and TnpPred [34], a tool to analyze prokaryotic transposases. Genome data and the resulting BADGE output are included in the BADGE repository for comparison and as an example. In addition, we compared the genomes of the brewery isolates with the genome of the winery isolate, as well as all genomes to each other in order to illustrate the shared gene content for all pairs of genomes. We also used BADGE to calculate the shared gene content of the five *P*. *damnosus* genomes and the *L*. *brevis* BSO 464 genome (BioProject PRJNA203088) [35]. In addition BADGE was used with two other data sets. Details about the used settings, the included genomes and the corresponding accession numbers can be found in S5 Table (*E*. *faecalis* example) and S6 Table (*B*. *amyloliquefaciens* example). Genomes were downloaded from the EBI and the NCBI.

### BLAST ring image

The BLAST ring image was generated with BRIG [36], while the blast output of BRIG was modified corresponding to BADGE default settings (95% percent identity, query / subject coverage 95%) using “awk” (AWK programming language, included in BASH).

### Evaluation of DMGs with PCR and statistical analysis

Using the fasta files containing all sequences of a given DMG of both SB strains and Clone Manager 9 (Scientific & Educational Software), sequences were aligned and consensus sequences were generated. Based on the consensus sequences, DMG specific primer pairs (S7 Table) were designed using Clone Manager 9. The *Taq* Core Kit 10 (MP Biomedicals) was used according to the manufacturer with 25 μl reaction volume, 0.5 μM primer and 0.75 U *Taq* DNA polymerase. Annealing temperature was set to 50°C for all DMG primers. Products were analyzed with agarose gel electrophoresis. In order to illustrate the suitability of DMGs (based on PCR evaluation) for the discrimination of beer spoilage ability and beer spoilage potential groups, we performed a statistical analysis. Spearman´s rank correlation tests for any monotone relationship between two variables and does not rely on normality. Spearman´s *ρ* and its significance were calculated regarding spoilage potential groups using R with the packages corrplot and Hmisc. Spearman´s *ρ* was interpreted according to Mukaka (2012) [37]. The goodness of DMGs with respect to spoilage ability was tested applying the two-sided Fisher´s exact test, using the R package stats.

## Results

### BADGE: BlAst Diagnostic Gene findEr

BADGE is focused on the prediction of DMGs and does not rely on laborious post-processing steps. The tool starts with fasta files containing the open reading frames (ORFs) and the genome sequences as input. The user is provided with files containing all necessary information about the potential DMGs as well as data for follow-up applications. For this purpose BADGE is using the well-known alignment toolkit of NCBI blast [32, 33], creating tabular output in combination with BASH commands in order to filter and compare the output. BADGE is available from https://github.com/TMW-bioinformatics/BADGE.

In default mode, optimized to predict DMGs for the discrimination of nearly related bacteria, BADGE predicts DMGs within six steps, as illustrated by Fig 1. In step 1 BADGE discards all ORFs of the target group A, which have a high quality megablast hit in the background group B-ORFs. In step 2 the remaining group A-ORFs (= A-DMGs) are compared to the group B genome sequences. This is done in order to identify A-DMGs having a high quality hit in B genomes, which were not discarded in step 1 as a consequence of frameshifts and inconsistent gene calling of genomes. These errors in gene calling result from sequencing errors and from automated genome annotations lacking manual curation. During step 3, the occurrence filter is applied to the remaining A-DMGs, retaining only those that are present in a chosen percentage of A-genomes. Setting the occurrence filter lower than 100% can be helpful to identify outliers or incorrectly assigned (e.g. wrongly grouped) strains. Two optional filters, at steps 4 and 5, discard those A-DMGs which have either long low-identity or short high-identity hits in the B genomes. This is done using dc-megablast and blastn, respectively.

The final output includes files containing the sequences of the A-DMGs in fasta format. One file contains a representative sequence for every predicted DMG. In addition, for each DMG one fasta file is created, containing all found sequences for the corresponding DMG. These files can be used directly for sequence alignment and consensus creation, thus enabling precise and correct primer design. Blast alignments in *html* format contain ‘residual’ alignments of A-DMGs to group B genomes. These are provided in order to get a visual and to most researchers familiar visualization of the DMG quality. A final tabular output file, which can be read with common spreadsheet applications, contains information about the quality, frequency and distribution of the DMGs in group A as well as their location. The user also gets access to all intermediate data, which offers full traceability (S8 Table).

BADGE results are affected by accuracy and consistency of gene calling. This is no problem if the improper annotation occurs in the genomes of the background group, as the BADGE genome filter handles this problem. However, if improper annotation is present within the genomes of the target group, a potential DMG might not be found, as a consequence of inconsistently predicted ORFs among this group. The included tool annotation_equalizer offers the possibility to equalize differences in annotations. The tool checks all genomes of user-defined groups for inconsistent gene predictions. ORF files containing the annotated ORFs of each genome are supplemented with alternative or missing gene calls, resulting from the comparison with the annotations of the other genomes of the group. Based on sequence similarity to ORFs in these other genomes, the corresponding regions are extracted from the genome files and added as extra ORFs to the ORF files. Using these files, BADGE is able to find potential DMGs within the target group, even if differences in ORF predictions exist.

By switching BADGE to “dc mode” (discontiguous megablast instead of megablast as basic algorithm for DMG prediction), the DMG finding process becomes slower but also more accurate. This is especially useful for the identification of potential DMGs having small deletions in the middle of a particular gene within the target group. In case of the megablast algorithm, depending on the size of a deletion, those variants result in two or more alignments, which prevent the identification of such genes as a potential DMG. DC-megablast extends the alignments when small gaps are present, resulting in the identification of those variants. BADGE can also be run in “protein level mode”, switching from megablast to blastp at step 1 and 3. Translation of ORFs is done automatically by the included program *fastatranslate* [38]. Accordingly the final output includes additional fasta files containing the corresponding amino acid sequences. In addition BADGE can be switched to “mut level” (mutation level) mode, either using DNA or amino acid sequences. This mode allows the identification of all identical gene sequences within group A, which differ from the corresponding sequences in group B in any form. This enables the prediction of potential single nucleotide polymorphism (SNP) or amino acid substitutions. We note, however, that the BADGE software was not explicitly developed for SNP detection. A BASH-tool called RAST2BADGE is included, modifying RAST annotated fasta files to become more”human readable”, informative and ready-made for a BADGE analysis. BADGE can also be used with fasta files obtained from the NCBI, EBI and other databases.

In case users want to compare a large number of genomes when the actual groups or phenotypes are not entirely clear or defined, one can make use of the BADGE distribution files. Simply use all your genomes as target group, create a dummy group containing a single dummy sequence fasta file (e.g. ATGCCC) as background group and run BADGE with a minimum DMG occurrence corresponding to one genome (e.g. 50 genomes, set to 0.02). BADGE will not discard any DMGs, but provide you with distribution files. These include the presence / absence of every gene within all included genomes. The distribution files can be further investigated using a spreadsheet application, e.g. applying the autofilter function, or they can be used for cluster analysis in order to define unknown groups.

BADGE was tested for seven popular unix distributions, including Linux and Mac OS systems. The installation of BADGE is very easy and quick. A supplementary manual is available, containing a detailed step-by-step guide for the installation of a virtual Linux system on Windows computers and subsequent BADGE installation, while the installation procedure for BADGE itself consists of download and extraction only. It is not necessary to compile anything nor do BADGE or the other binaries rely on complex dependencies. Test data sets are provided as well as a tool to check for a correct ‘installation’ of BADGE. Uninstallation of BADGE is achieved by deleting the BADGE folder. All settings and filters can be changed easily with any text editor, while each run of BADGE creates a *settings*-file for future purposes. For a short description of all settings see S3 Table. Copies of BADGE can be created to run multiple instances of BADGE in parallel. The execution time of BADGE, using parallel blast processes, depends on the number and size of genomes to be compared and the available computing resources. The prediction of 66 DMGs for the discrimination of beer spoiling strains from non-spoilers of *P*. *damnosus* took 3 min on a standard personal computer. In this analysis, two group A genomes were compared with three group B genomes, all having an approximate genome size of 2.3 Mbp.

BADGE was tested for its capability to predict DMGs using different data. We compared the genomes of two clinical isolates of *Enterococcus faecalis* with a probiotic strain, using BADGE with default settings (for details see S5 Table). BADGE predicted 56 DMGs for the clinical isolates, including the important virulence associated genes *gelE*, *fsrB* and *sprE* [39, 40]. By PCR screening, these genes were also found to be only present in both clinical isolates in a previous study [41]. In addition we predicted DMGs which are specific for *Bacillus amyloliquefaciens* subsp. *plantarum* and not present in *B*. *amyloliquefaciens* subsp. *amyloliquefaciens*, while these subspecies are characterized by a different lifestyle [42]. Genome comparison was conducted with 10 genomes of the plant associated subspecies *B*. *plantarum* and four genomes of *B*. *amyloliquefaciens*, as done in a previous study [42]. Using BADGE with default settings, only parts of the important *B*. *plantarum* specific gene clusters for the synthesis of the secondary metabolites macrolactin (*mln* cluster) and difficidin (*dfn* cluster) were found. This resulted from inconsistent annotation of two genomes within the 10 investigated *B*. *plantarum* genomes. After using the optional tool annotation_equalizer, all genes of both genetic clusters were identified in all 10 genomes. In total, 158 DMGs were predicted, as shown in detail in S6 Table. Finally, we tested BADGE with five novel *P*. *damnosus* genomes, predicting DMGs for the differentiation of beer spoiling and non-spoiling strains, which is described in the next sections.

### Evaluation of beer spoilage potential and genome sequencing of *P*. *damnosus*

All strains of *P*. *damnosus* were classified into four beer spoilage groups ranging from no spoilage potential to strong spoilage potential. The classification depends on their ability to grow in beers with increasing antibacterial stability. Table 1 lists all strains, their source and their classification into one of the spoilage potential groups. Strains with middle to strong spoilage potential are considered as beer spoiling strains (= beer spoilage ability). The transferability and stability of the resulting classification into spoilage potential groups was tested for four strains, using beers of alternative breweries. All four strains showed a consistent spoilage potential within all beer sets tested. Five strains, four of them being brewery isolates and one of them isolated from a winery environment (W), were chosen for whole-genome sequencing using single molecule real time (SMRT) sequencing, which is also marked in Table 1. Two of the chosen strains showed a strong beer spoilage potential (SB), while three of them were not able to grow in any of the tested beers, thus deemed to have no spoilage potential (NB). After *de novo* assembly with SMRT Analysis, genomes were annotated with the Rapid Annotations using Subsystems Technology [30, 31] and completed running RAST2BADGE. S2 Table shows important metrics of sequencing, assembly and annotation for all five genomes.

### Genome analysis and the identification of novel DMGs for the discrimination of beer spoiling *P*. *damnosus* strains from non-spoiling strains using BADGE

Five complete (closed) genomes were generated. The chromosome size ranges from 2.07 to 2.25 Mbp with a GC content of 38.2 to 38.9%. Starting with two plasmids in case of the winery isolate, four to seven plasmids were found for the brewery isolates with GC contents from 37.3 to 43.3%. Plasmid size ranges from 7,678 bp (TMW 2.1536-NB-W) to 143,870 bp (TMW 2.1535-SB), the latter which can be considered as a megaplasmid. In case of the beer spoiling strains more than 10% of the genome is plasmid encoded, resulting in 2.40 to 2.51 Mbp genome sizes. In comparison, the non-spoiling strains have whole genome sizes ranging from 2.17 to 2.28 Mbp. In total 2,017 to 2,330 protein encoding genes were found, resulting in a coding density of about 84%, while the core genome contains 1,580 gene families and the pan genome 2,374, calculated with CMG biotools [22]. Consequently the accessory genome makes up 19 to 32%. Four complete rRNA operons, 60 tRNAs and two pseudo-tRNAs were found.

**Fig 2:**
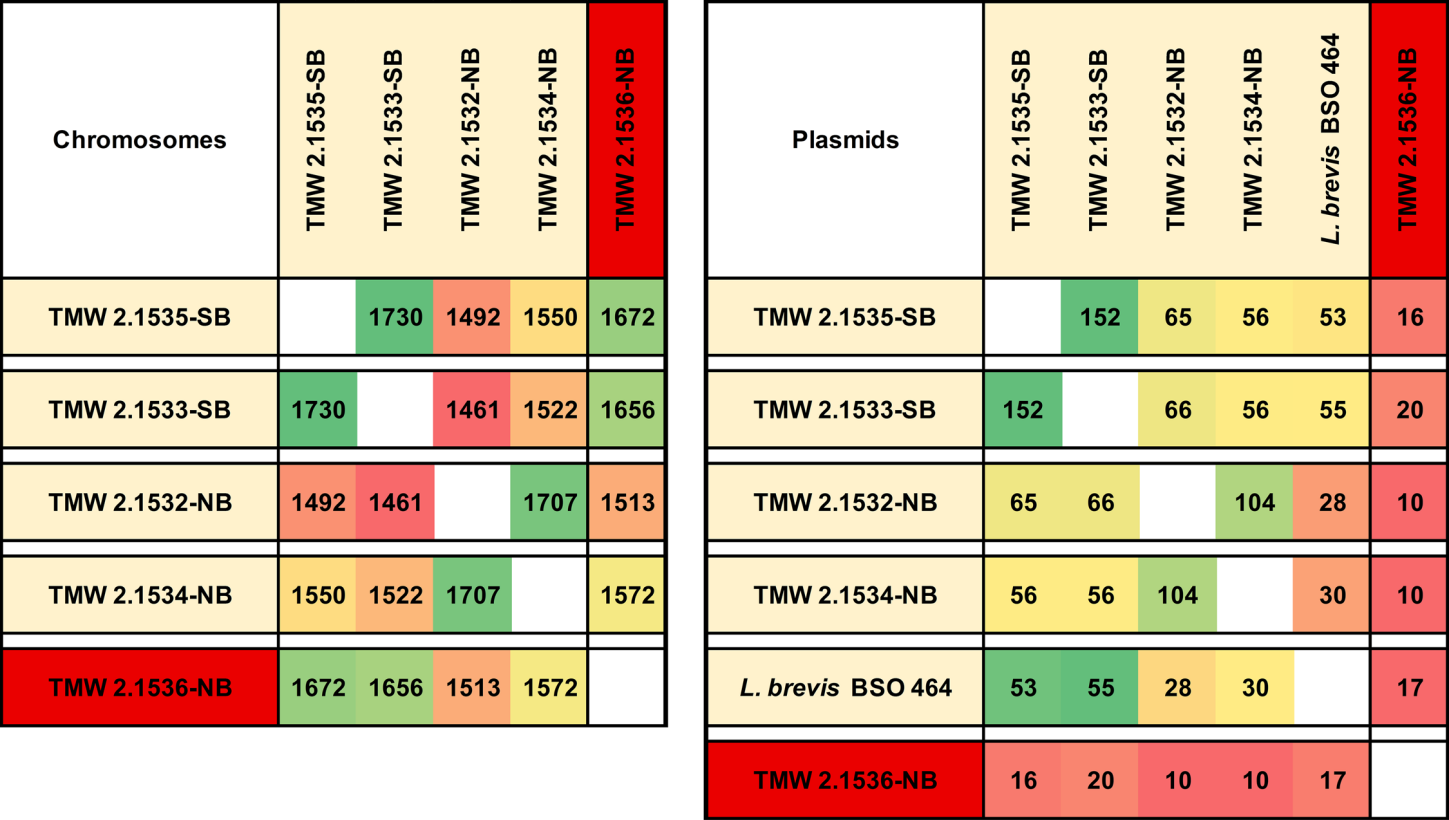
Shared gene content of *P*. *damnosus* genomes. The number of genes shared between strains is shown for each combination, once for all chromosomal and once for all plasmid encoded genes. In case of the plasmid comparison, we included the plasmidome of the rapid beer spoiling *L*. *brevis* BSO 464 [35]. Brewery strains and the winery isolate TMW 2.1536-NB are color labeled for differentiation. Shared genes were predicted using BADGE with default settings. NB = no spoilage potential, SB = strong spoilage potential according to beer spoilage test.

With BADGE in default mode, we compared the five genomes in order to predict brewery specific and spoilage group specific DMGs. Fig 2 shows the amount of shared genes for all possible combinations, illustrating that brewery specific DNA is mainly found on plasmids. We identified 1025 genes which were only present in brewery isolate genomes. 205 were shared by at least two brewery isolate genomes, while 33 of them were found in all four brewery strains. 30 of these genes are plasmid encoded and include genes related to biotin uptake, fatty acid modification and alpha-acetolactate metabolism. The alpha-acetolactate cluster encodes for acetolactate synthase (EC 2.2.1.6) and alpha-acetolactate decarboxylase (EC 4.1.1.5). The former enzyme is responsible for the decarboxylation of pyruvate to acetolactate, which is spontaneously oxidized to the off-flavour diacetyl. These enzymes are neither encoded chromosomally, nor found within the whole genome of the winery isolate TMW 2.1536-NB. Further these shared genes include the *horA* and the *horC* cluster. We also calculated the plasmid encoded shared gene content of *P*. *damnosus* to the plasmids of the rapid beer spoiling strain *L*. *brevis* BSO 464 [35]. The amount of shared genes is highest for both beer spoiling *P*. *damnosus* and lowest for the winery isolate, TMW 2.1536-NB.

**Fig 3:**
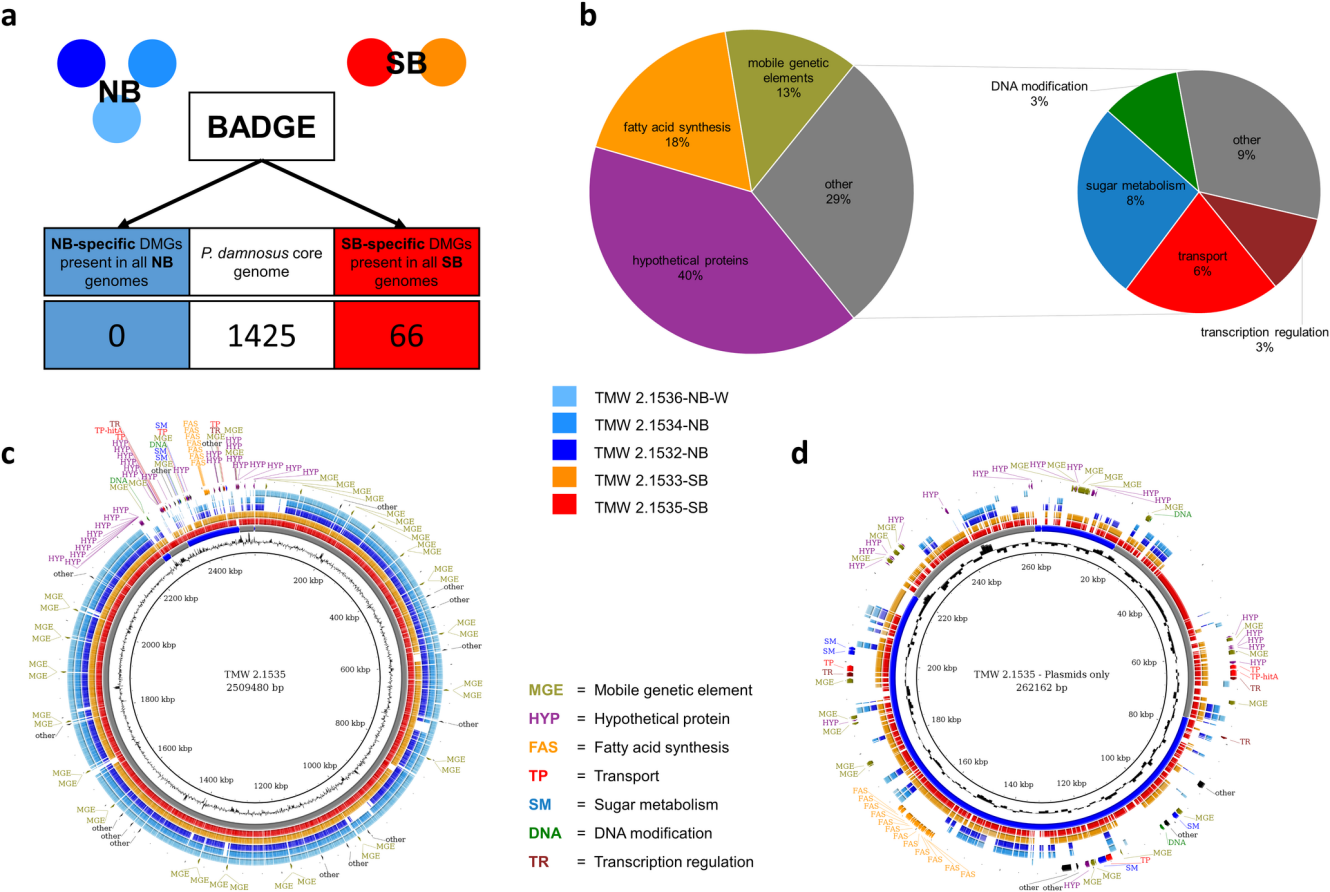
Visual illustration of BADGE results of the *P*. *damnosus* beer spoilage example. (a) Illustration of BADGE comparison of the group of beer spoiling strains with the group of non-spoiling strains. The number of genes, shared by all five strains is also shown. All calculations were done with BADGE using default settings. (b) Percentage of SB-DMGs regarding their function based on RAST annotation. (c) BLAST ring image for whole genomes of all five strains. All rings are described from the inside to the outside: ring 1 (black) shows the whole genome of TMW 2.1535-SB as reference with bp coordinates; ring 2 (black) shows the GC content of TMW 2.1535-SB; ring 3 consists of arcs with different lengths and alternating coloration (grey / blue) representing the contigs (chromosome and plasmids) of TMW 2.1535-SB; ring 4 (red) shows all blast hits of TMW 2.1536-SB ORFs versus its own genome illustrating the coding density; ring 5 (orange) shows all blast hits of TMW 2.1533-SB ORFs versus the reference; rings 6–8 (blue shades) show all blast hits of the NB strains ORFs versus the reference, ring 9 contains all DMGs predicted by BADGE mapped to the reference, located in the gaps of the rings 6–8. Note that a particular DMG may occur more than once in a genome. Therefore the number of DMG labels does not correspond to the number of DMGs calculated by BADGE. (d) BLAST ring image for plasmids only. Most DMGs are located within clusters, e.g. in a cluster containing genes for fatty acid synthesis (FAS). For descriptions of the rings see (c).

More important, BADGE predicted 66 potential DMGs, which were present only in both strong spoilage strains (target group) and absent in all non-spoiling strains (background group). As already described, BADGE output can be affected by gene calling and the consistency of annotation. Thus the same comparison was also conducted with genes predicted by glimmer [43–45]. A comparison of both outputs revealed a 7.6% difference in DMG prediction, which could be reduced to 3.8% by applying the annotation_equalizer. Mainly very small and hypothetical proteins were found to be different. Using a spreadsheet application with the final tabular output file (S4 Table), functional and spatial gene clusters could be identified. Fig 3C and 3D show the localization of all DMGs using BRIG, illustrating that more than 95% of all potential DMGs are located on plasmids. Most DMGs are potentially encoding for hypothetical proteins, are part of a fatty acid synthesis cluster or mobile genetic elements (Fig 3B). Based on function, quality and localization of the predicted DMGs, 10 candidates were selected for primer design. For designation and predicted function see Table 3.

**Table 3:** Designation of BADGE predicted diagnostic marker genes (DMGs) and predicted function (annotation). All listed genes were found to be present in both beer spoiling genomes and absent in the non-spoiling genomes. If RAST annotation differs from blastp (vs. nr database) result, both are given.

DMG designation	locus tag TMW 2.1533	locus tag TMW 2.1535	annotation (RAST / blastp)
***parA***	ADU70_0027	ADU72_2395	plasmid partitioning protein
***tetR***	ADU70_0142	ADU72_2392	transcription regulator (TetR family)
***tnpA***	ADU70_0196	ADU72_2387	transposases (IS30 family)
***fabZ***	ADU70_0261	ADU72_0122	3-hydroxyacyl-[acyl-carrier-protein] dehydratase
***icaA***	ADU70_0037	ADU72_0055	intercellular adhesion protein / polysaccharide deacetylase
***nplA***	ADU70_0212	ADU72_0137	neopullulanase
***galM***	ADU70_0186	ADU72_0163	aldose 1-epimerase
***hypA***	ADU70_0277	ADU72_0017	hypothetical protein
***tnpB***	ADU70_0197	ADU72_2388	transposases (IS30 family)
***npxA***	ADU70_0144	ADU72_0045	NADH peroxidase

**Fig 4:**
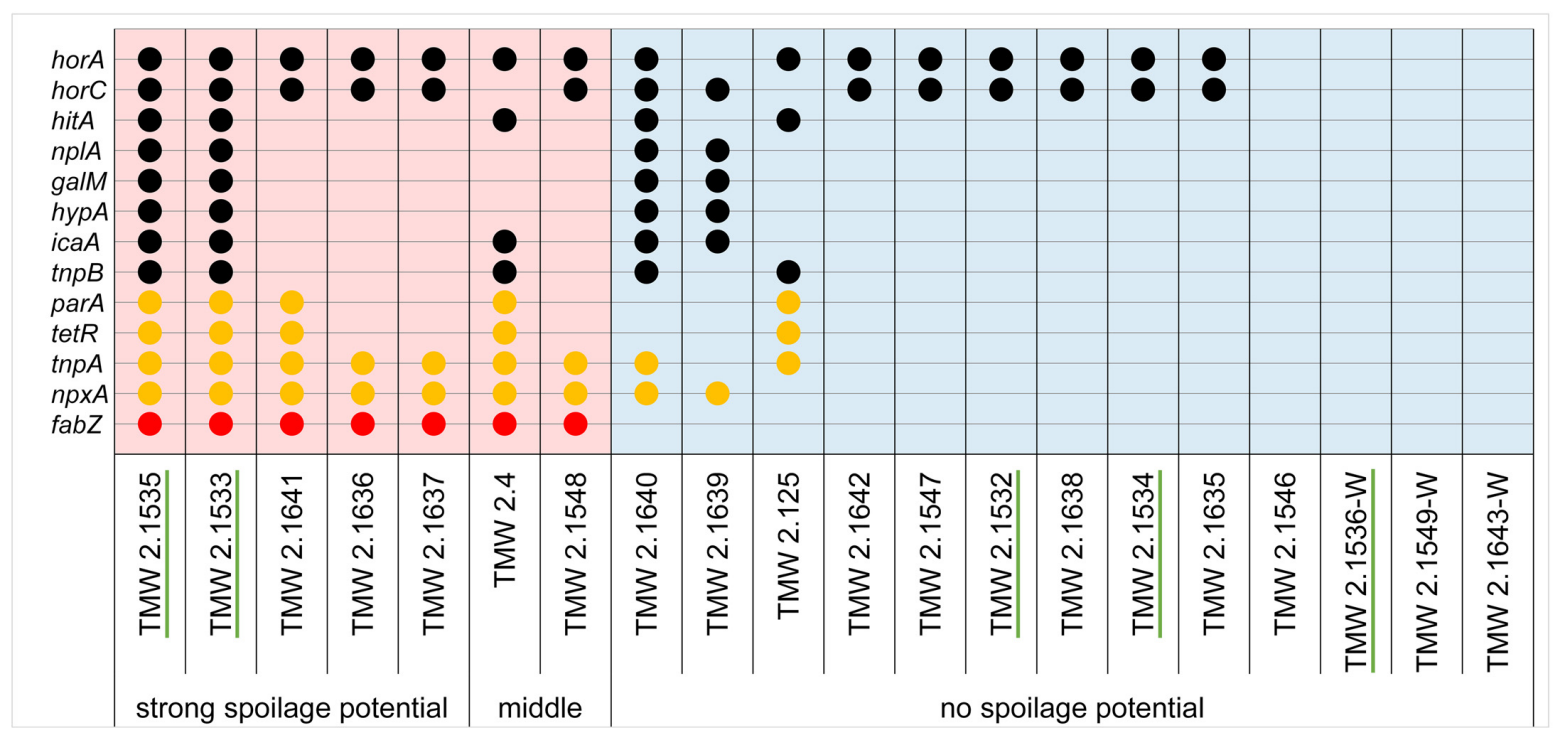
DMG evaluation and validation. 20 strains of *P*. *damnosus* were tested for the presence of the predicted DMGs and already published DMGs using PCR. Note that the published DMGs *horA* and *horC* were not predicted by BADGE, as both genes are present in two of three non-spoiling genomes. Still, all strains were tested for their presence and results are shown for comparison. A positive reaction is indicated with a dot. Significant correlation to spoilage potential is indicated using the colors red (very high) and orange (moderate to high). Genome sequenced strains are underlined.

Fasta files containing all sequences of each chosen DMG were further processed with Clone Manager 9. DMG sequences were aligned and consensus sequences were generated. Based on the consensus sequence, DMG specific primer pairs were designed (S7 Table). These primers, as well as primers for the published DMGs *horC*, *horA and hitA* for comparison, were used for PCR evaluation and validation. 15 additional strains of *P*. *damnosus* with known spoilage potential were tested. Fig 4 illustrates the PCR results and shows that 5 of the 10 chosen DMGs are useful for the discrimination of beer spoiling and non-spoiling strains of *P*. *damnosus*. Table 4 shows the results of Spearman´s rank correlation and Fisher´s exact test for the tested DMGs. *ParA*, *tetR*, *tnpA*, *npxA* and especially *fabZ* show a significant relation to beer spoilage potential and spoilage ability. The established marker genes *horC*, *horA* and *hitA* did not show a significant relation to both classifications.

**Table 4:** Statistical analysis of DMG evaluation. Spearman´s rank correlation to beer spoilage potential as well as Fisher´s P (two-sided) with respect to beer spoilage ability are listed in order to illustrate the quality of the tested DMGs. The percentage of correct identifications is listed for each DMG, based on the assumption that each DMG is made for the discrimination of beer spoiling and non-spoiling strains (beer spoilage ability). Further the fractions of false positive and false negative results are given, also regarding the detection of beer spoilage ability. DMGs are sorted descending according to Fisher´s P. NS = Spearman´s ρ not significant at CI = 95%.

DMG designation	Fisher´s P	Spearman´s ρ	Correct discrimination (%)	False positive (x/13)	False negative (x/7)
***nplA***	0.59	NS	65	2/13	2/7
***galM***	0.59	NS	65	2/13	2/7
***hypA***	0.59	NS	65	2/13	2/7
***horC***	0.35	NS	55	8/13	1/7
***hitA***	0.29	NS	70	2/13	4/7
***icaA***	0.29	NS	70	2/13	4/7
***tnpB***	0.29	NS	70	2/13	4/7
***horA***	0.11	NS	60	8/13	0/7
***parA***	0.031	0.55	80	1/13	3/7
***tetR***	0.031	0.55	80	1/13	3/7
***tnpA***	0.00046	0.79	90	2/13	0/7
***npxA***	0.00046	0.79	90	2/13	0/7
***fabZ***	0.00001	0.98	100	0/13	0/7

*FabZ* is part of a plasmid encoded complete fatty acid biosynthesis (FAS) cluster, while all sequenced *P*. *damnosus* strains, regardless of isolation source and spoilage potential, were found to have an incomplete chromosomal fatty acid biosynthesis. These chromosomal genes encode for acetyl-CoA-carboxylase (*accABCD*, EC 6.4.1.2), acyl carrier protein (*accP*), malonyl-CoA-acyl-carrier-protein-transacylase (*fabF*, EC 2.3.1.39), 3-oxoacyl-acyl-carrier-protein-reductase (*fabG*, EC 1.1.1.100) and 3-oxoacyl-acyl-carrier-protein-synthase III (*fabH*, EC 2.3.1.180), while they do not encode for 3-hydroxyacyl-acyl-carrier-protein-dehydratase (*fabZ*, EC 4.2.1.59) and enoyl-acyl-carrier-protein-reductase I (*fabI*, EC 1.3.1.9), both which are necessary enzymes for fatty acid chain elongation. In contrast, the plasmid encoded FAS cluster, exclusively found within strong spoiling strains, encodes all of the abovementioned enzymes and proteins. Blast analysis of the plasmid encoded FAS cluster revealed no similarity on DNA level to those encoded on the chromosome (no dc-megablast hits). Further, we did not identify high similarities (above 73%) to any sequences within the NCBI nr/nt database. So far, this specific FAS cluster was found exclusively in brewery isolated *P*. *damnosus* strains. In order to demonstrate the importance of fatty acids for *P*. *damnosus* growth in beer, we conducted a modified beer spoilage test, where we examined the ability of non-spoiling strains to grow in lager beer 1 with and without a source of additional fatty acids. Table 5 shows that non-spoiling brewery isolates are capable of spoiling beer when supplemented with fatty acids. The non-spoiling winery isolate TMW 2.1643-NB was neither able to grow in lager beer 1 with a fatty acid source, nor without the addition.

**Table 5:** Modified beer spoilage test—growth in lager beer 1 with and without an additional fatty acid source. Lager beer 1 with and without an additional fatty acid source was inoculated with non-spoiling and spoiling strains. Genome sequenced strains are underlined. TMW 2.1535-SB served as positive control. Four non-spoiling strains were tested for their ability to grow in lager beer 1 with an additional fatty acid source, one of them sequenced and three which were tested for *fabZ* using PCR. Non-spoiling (*fabZ* negative) brewery isolates were capable of growing in lager beer 1 supplemented with an additional fatty acid source. Spoilage-groups derived from original beer spoilage test: strong beer spoilage potential (SB)—growth in pilsner, middle potential (MB)—growth in lager beer, weak potential (WB)—growth in wheat beer, no potential (NB)—no growth in test beers. Brewery as isolation source, the presence of *fabZ* and growth in test beers are indicated by +. IBU = international bitterness units (measure for hop content).

Strain	Spoilage category	Brewery isolate	*fabZ*	Growth in lager beer 1, pH 4.3, 18 IBU, 5.1 vol. % alcohol	Growth in lager beer 1 + fatty acids
TMW 2.1535	SB	+	+	+	+
TMW 2.1532	NB	+	-	-	+
TMW 2.125	NB	+	-	-	+
TMW 2.1639	NB	+	-	-	+
TMW 2.1643	NB	-	-	-	-

## Discussion

### BADGE–a use-oriented solution for the prediction of DMGs

BADGE is a free tool, which, in combination with easy-to-use and intuitive applications for genome assembly (e.g. SMRT Analysis) and annotation (e.g. RAST), will help to make comparative genomics available to a broad user group as a means to an end. In our application example we demonstrated the functionality of BADGE to predict DMGs using NGS data. Within a few minutes two groups of pediococci, comprising five genomes, could be compared with regard to differentiating gene sequences. In contrast to other genome comparison tools, BADGE result-tables and -sequences are focused on providing only needed information for immediate evaluation and selection of appropriate diagnostic marker genes (DMGs), discriminating the defined phenotypic groups. The distribution of DMGs as well as the frequency within single genomes can be directly accessed and evaluated. Subsequent applications are supported by fasta files containing all sequences for each DMG, which can be used directly for consensus sequence generation and primer design.

Since BADGE looks for discriminatory genes instead of sequence fragments, it relies on the consistency and quality of gene calling and annotation. Potential DMGs might not be found if they are not or inconsistently annotated within the target group, while missing annotations in the background group have no impact. The probability of such an event can be decreased by using consistently annotated genomes, as for example done by the NCBI Prokaryotic Genome Annotation Pipeline or RAST [30, 31, 46]. In addition we provide the tool annotation_equalizer, which is able to reduce the described impact to a minimum. Annotation_equalizer identifies inconsistent ORF predictions and equalizes these inconsistencies within the included genomes by modifying the respective fasta files. This way originally non- or differently annotated genes and pseudogenes can be included into BADGE analysis. Further, BADGE was primarily developed for complete genomes, but was also tested successfully with draft genomes. The inclusion of incomplete genomes may cause false positive predictions (incomplete genomes in background group) or result in a reduced number of predicted DMGs (incomplete genomes in target group), while the latter effect can be decreased by reducing the minimum DMG occurrence. However, the quality of genomes and the assembly level affect all kinds of comparative analyses aiming to find differentiating sequence information. As the costs for long read sequencing will decrease, the proportional abundance of incomplete bacterial genomes will also be decreasing, reducing negative effects of missing information for all kinds of genome analyses.

The major advantage of BADGE is the strict design for simplicity of use compared to extensive genome comparison tools [21–23]. BADGE is specifically designed for DMG prediction, unlike other tools with a different focus. BADGE is a single BASH script, which is executed from command line or double clicked and no additional dependencies need to be installed. Therefore, it uses as few commands as necessary for functionality in order to keep the code compact and portable. The DMG prediction can be set to three selectable methods and a few associated changeable settings and optional filters. During the BADGE run the user is informed how many DMGs are passing the applied filters step by step. This makes it easy to evaluate the impact of chosen settings and options. Accordingly BADGE is not intended to be a genome comparison black-box providing a mass of (unused) data and graphs. Instead, its purpose is it to enable the end-user to understand the underlying DMG prediction mechanism and conduct alterations to the user´s needs. The use of a simple BASH script, instead of a graphical interface based application, provides experienced programmers the possibility to quickly modify the code according to their own criteria. It also enables first time command line users a possibility to easily familiarize with the BADGE script. BADGE is simple to install and use and it avoids laborious data interpretation and sequence extraction. BADGE is a straightforward copy and paste tool to a diagnostic marker gene PCR assay, while it offers full flexibility in parametrization and coding.

#### The identification of *fabZ*, a novel and outstanding DMG for the discrimination of beer spoiling and non-spoiling *P*. *damnosus* strains

For a fast and reliable PCR based detection method for beer spoiling *P*. *damnosus*, DMGs must have a significant correlation to beer spoilage potential and beer spoilage ability. We classified 20 strains into beer spoilage groups and could also show that the resulting classification, and therefore also a potential DMG differentiating these groups, is transferable to other beers. Published DMGs (*horA*, *horC*, *hitA*) for the differentiation of beer spoiling and non-spoiling lactic acid bacteria did not show a significant correlation to the beer spoilage potential of 20 strains of *P*. *damnosus*. In contrast, 5 of 10 BADGE-predicted DMGs showed moderate to very high positive correlation. The gene *fabZ*, which encodes for a 3-hydroxyacyl-acyl-carrier-protein-dehydratase (EC 4.2.1.59), was shown to be an outstanding DMG for the discrimination of beer spoiling and non-spoiling strains. All three sequenced non-spoiling strains lack *fab*Z and have a chromosomal encoded incomplete type II fatty acid biosynthesis pathway. In contrast, both beer spoiling strains have an additional plasmid encoded complete type II fatty acid biosynthesis cluster. In addition, PCR validation showed that only *fabZ* positive strains were able to spoil beer. This and the fact that non-spoiling brewery strains were able to grow in lager beer with an additional fatty acid source, but not in unmodified lager beer, indicates that a complete fatty acid biosynthesis pathway is required for *P*. *damnosus* strains to grow in beer. This is in accordance with previous studies where the relevance of membrane biosynthesis and modification as a passive hop tolerance mechanism for growth in beer was indicated [47, 48]. In contrast to *horC* or *hitA*, *fabZ* does not represent a brewery (spoilage) specific gene, encoding a specific, e.g. hop resistance related function. A 3-hydroxyacyl-acyl-carrier-protein-dehydratase (*fabZ*) is found in various bacteria with the ability to synthesize fatty acids *de novo*. Spoilage and brewery specific is that specific piece of DNA encoding *fabZ* and the attendant FAS cluster. Since *P*. *damnosus* is not equipped with a stable, chromosomal encoded FAS biosynthesis, the plasmid encoded *fabZ* (FAS cluster) can be considered as a true lifestyle gene, enabling the growth in the specific ecological niche beer.

#### Beer spoilage ability–a plasmid encoded trait?

Brewery isolated *P*. *damnosus* genomes are characterized by a high amount of plasmid encoded information, representing up to 10.6% of their total DNA in case of the beer spoiling strains. Within the genus *Lactobacillus*, we could only find a higher ratio of plasmidome to genome size for strains (complete genomes) of *Lactobacillus salivarius*. *L*. *salivarius* plasmids are considered to contribute to niche adaption, in this case the mammalian gastrointestinal tract [49]. The number of *P*. *damnosus* plasmids increases with beer spoilage potential, while both sequenced strong spoiling strains also share more genes than they share with non-spoiling strains. The shared gene content is also related to the isolation source, while brewery isolates are characterized by brewery enriched or brewery specific plasmid encoded genes. This trend is even confirmed on an interspecies level, as *L*. *brevis* BSO 464 also shares most plasmid genes with both sequenced *P*. *damnosus* beer spoiling strains, followed by the other brewery isolates and finally the winery isolate TMW 2.1536-NB. Thus, there is pool of brewery specific DNA, including important hop resistance genes as *horA*, *horC* and *hitA*. However, this pool of DNA is not restricted to these prominent DMGs and it has been shown, that the presence of plasmids lacking these genes also has an impact on the growth of *L*. *brevis* BSO 464 in beer [50]. In case of *L*. *brevis* BSO 464, it was demonstrated that the loss of the *horC* bearing plasmid pLb464-2 has the most dramatic (negative) effect on hop tolerance and growth in beer, while two other plasmids were also found to support the strains growth in beer. As *L*. *brevis* BSO 464 encodes a complete chromosomal fatty acid biosynthesis, there is no need for an additional plasmid encoded FAS cluster, which is also true for the beer spoiling strain *Pedicoccus claussenii* ATCC BAA-344, again characterized by a high number of plasmids and brewery specific DNA [51]. While the presence of *horC* seems to be crucial for *L*. *brevis* BSO 464 growth in beer, this role seems to be reserved for the FAS cluster in case of *P*. *damnosus*. This does not mean that *horC*, *horA*, *hitA* or any other not yet characterized gene found on these brewery plasmids have no significance for *P*. *damnosus* beer spoilage ability, but it rather indicates that these genes are of subordinate significance, in an hierarchical way. It is even likely that both, the FAS cluster and hop resistance genes like *horC*, are needed for successful growth in beer, which is supported by the inability of the winery isolate TMW 2.1643-NB, lacking *fabZ*, *horC*, *horA* and *hitA*, to grow in beer with an additional fatty acid source. Thus, our results confirm and extend the findings of a previous study of Bergsveinson and coworkers. LAB growth in beer is a multifactorial process, relying on the presence of plasmid encoded genes. While the presence of some specific plasmids and genes seems to be a prerequisite for bacterial growth in beer, others might contribute to increased growth success in beer [50]. We found that the ability to produce the unwanted off-flavor diacetyl (indirectly) from pyruvate is a plasmid encoded trait, while the corresponding alpha-acetolactate operon was only found in case of the brewery isolates. This indicates that this metabolic capability is not only troubling brewers, but also represents a metabolic advantage for *P*. *damnosus* in beer. This makes sense, if it is considered that beer in general has a low pH of 3.8 to 4.7 [13]. As the homofermentative metabolism of *P*. *damnosus* normally results in lactate production, the production of non-acidic end products in an intrinsically acidic environment might be an advantage. Nevertheless, lacking a respiratory chain, *P*. *damnosus* has to recycle its NAD^+^ using alternative electron acceptors, as this would normally happen during lactate production. Therefore it is interesting that both sequenced beer spoiling strains encode a chromosomal acetoin reductase (EC 1.1.1.4), allowing the subsequent reduction of acetoin to 2,3-butanediol, thus recycling NADH. The winery isolate genome of TMW 2.1536-NB was found to also encode for an acetoin reductase, while we could not identify the genes encoding for diacetyl and acetoin production.

## Conclusions

BADGE is an easy-to-use and fast comparative genomics tool to answer the simple but often asked question: How can I differentiate my bacteria at the gene level and which genes are responsible for a specific phenotype? We show a straightforward procedure, from phenotyping, genome assembly as well as annotation and the usage of BADGE up to a diagnostic marker gene PCR assay, making comparative genomics a means to an end. BADGE and the established procedure were successfully evaluated using an example with high relevance for the brewing industry. We identified a novel outstanding diagnostic marker gene (*fabZ*) for the identification of beer spoiling *P*. *damnosus*, which can be applied for spoilage risk determination in breweries. Our findings also indicate that a complete fatty acid biosynthesis is required for *P*. *damnosus* in order to grow in and consequently spoil beer. Further, we found that the ability of *P*. *damnosus* to produce acetolactate from pyruvate, and therefore the formation of the well-known and unwanted off-flavor diacetyl, is a plasmid encoded trait, which might contribute to a better adaption of this organism to beer.

## Supporting Information

S1 Table Results of beer spoilage test. In addition to beer spoilage potential category (spoilage-groups: strong beer spoilage potential (SB)—growth in pilsner (29 IBU). middle potential (MB)—growth in lager beer (18 IBU). weak potential (WB)—growth in wheat beer (14 IBU). no potential (NB)—no growth in test beers). OD_590_ and pH after 60 days of incubation are listed.
(DOCX)

S2 Table Genome metrics for sequenced strains. Genomes result from single molecule real time sequencing (Pacific Biosciences) and consequent hierarchical genome assembly process. Resulting contigs were manually curated and circularized. Annotation was performed using Rapid Annotations using Subsystems Technology (RAST) in default settings and RAST2BADGE. All biosamples are part of the bioproject PRJNA290141. Annotated features contain protein encoding genes as well as RNA features. TMW = Technische Mikrobiologie Weihenstephan.
(DOCX)

S3 Table BADGE settings with input range, default value und description. Changing BADGE settings can be easily done using any text editor. Open *BADGE*.*sh* with your text editor and change settings as desired. Save your changes before you start a new BADGE run. We recommend to save a copy of *BADGE*.*sh* somewhere else. If your version does not work anymore (e.g. due to a typo), just replace it by the original *BADGE*.*sh* script.
(DOCX)

S4 Table Tabular output of BADGE in default setting using the *P*. *damnosus* genomes. DMG_ID = unique identifier assigned to every DMG; percent_occurrence: proportion (0–100%) of target group (spoilers) genomes a DMG is present in; dc_blast_hit: yes / no, yes if a DMG has a remaining dc-megablast hit (with quality values below those selected in BADGE) within the ‘opposed’ group (non_spoilers); max_blastn: the maximum length of a blastn hit produced by a DMG in the ‘opposed’ group (non_spoilers); ORF_ID: Identifiers of all existing ORFs of the DMG (ORF IDs for each strain, if frequency of ORFs of the DMG within a genome is higher than 1 (multiple copies of a gene within the genome), more than 1 ID per strain is possible); ORF_length: length of ORFs; annotation: contains information about the predicted function (depends on genome annotation); contig: names the contig where the ORF is located (plasmid, chromosome); start / stop: coordinates on contig in base pairs (NOTE: if frequency of ORFs of the DMG within a genome is higher than 1 and members of a DMG are exactly identical, single members cannot be differentiated within one genome and consequently all positions (start-stop) of the gene with multiple copies are given with ascending coordinates).
(XLSX)

S5 Table Tabular output of BADGE in default setting using 3 *Enterococcus faecalis* genomes. 2 clinical isolates (*E*. *faecalis* OG1RF—CP002621, *E*. *faecalis* V583—AE016830) were compared to one probiotic strain (*E*. *faecalis* Symbioflor 1—HF558530). Genomes were obtained from the EBI. The table shows the DMGs predicted for the clinical isolates, including known virulence factors *gelE* (DMG_17), *fsrB* (DMG_19) and *sprE* (DMG_16). DMG_ID = unique identifier assigned to every DMG; percent_occurrence: proportion (0–100%) of target group genomes a DMG is present in; dc_blast_hit: yes / no, yes if a DMG has a remaining dc-megablast hit (with quality values below those selected in BADGE) within the ‘opposed’ group; max_blastn: the maximum length of a blastn hit produced by a DMG in the ‘opposed’ group; ORF_ID: Identifiers of all existing ORFs of the DMG (ORF IDs for each strain, if frequency of ORFs of the DMG within a genome is higher than 1 (multiple copies of a gene within the genome), more than 1 ID per strain is possible); ORF_length: length of ORFs; annotation: contains information about the predicted function (depends on genome annotation); contig: names the contig where the ORF is located (plasmid, chromosome); start / stop: coordinates on contig in base pairs. (NOTE: if frequency of ORFs of the DMG within a genome is higher than 1 and members of a DMG are exactly identical, single members cannot be differentiated within one genome and consequently all positions (start-stop) of the gene with multiple copies are given with ascending coordinates).
(XLSX)

S6 Table Tabular output of BADGE in modified settings using 14 *Bacillus amyloliquefaciens* genomes, comprising 2 different subspecies. Settings were changed at two positions: megablast_perc_identity_cut = 90, dc_perc_identity_cut = 85. Strains of *Bacillus amyloliquefaciens subsp*. *amyloliquefaciens*: DSM 7—FN597644, LL3—CP002634, TA208—CP002627, XH7—CP002927. *Bacillus amyloliquefaciens subsp*. *plantarum*: AS43.3—CP003838, CAU B946—HE617159, CC178—CP006845, FZB42—CP000560, IT-45—CP004065, NAU-B3—HG514499, UCMB5036- HF563562, UCMB5113—HG328254, Y2—CP003332, YAU B9601-Y2—HE774679. The table shows the DMGs predicted for the subspecies *B*. *plantarum*, including all genes of the important genetic clusters for synthesis of the secondary metabolites macrolactin and difficidin. mlnABCDEFGHI: DMG_37—DMG_45. dfnAYXBCDEFGHIJKLM: DMG_71—DMG_85. DMG_ID = unique identifier assigned to every DMG; percent_occurrence: proportion (0–100%) of target group genomes a DMG is present in; dc_blast_hit: yes / no, yes if a DMG has a remaining dc-megablast hit (with quality values below those selected in BADGE) within the ‘opposed’ group; max_blastn: the maximum length of a blastn hit produced by a DMG in the ‘opposed’ group; ORF_ID: Identifiers of all existing ORFs of the DMG (ORF IDs for each strain, if frequency of ORFs of the DMG within a genome is higher than 1 (multiple copies of a gene within the genome), more than 1 ID per strain is possible); ORF_length: length of ORFs; annotation: contains information about the predicted function (depends on genome annotation); contig: names the contig where the ORF is located (plasmid, chromosome); start / stop: coordinates on contig in base pairs. (NOTE: if frequency of ORFs of the DMG within a genome is higher than 1 and members of a DMG are exactly identical, single members cannot be differentiated within one genome and consequently all positions (start-stop) of the gene with multiple copies are given with ascending coordinates; overlapping DMGs are indicated in the last column).
(XLSX)

S7 Table DMG specific primer pairs. Primer sequences were created from consensus sequences of all available open reading frames of a given DMG. PCR was performed at 50°C for all marker genes. *horA*, *horC* and *hitA* are DMGs which were not identified within this study.
(DOCX)

S8 Table Description of all files produced by BADGE in verbose mode. Description of all files produced by BADGE in verbose (clean_up = false) mode (group A genomes/ORFs were named A1 and A2, group B genomes/ORFs were named B1, B2 and B3). In case of using PROTEIN-LEVEL BADGE additional, analog designations are created with amino acid sequences. If the filters of Step 4 and / or 5 are disabled, the corresponding files are not produced. The term “blast hit” within the descriptions refers to a blast hit based on the chosen settings for a particular blast. The files which allow the user to easily follow the DMG finding process are printed bold (note: open files with file name extension:.distribution,.frequency and.tsv with spreadsheet application; open files with file name extension:.alignment with web browser; all other files can be opened with any text editor). Box highlights files directly suited for follow-up applications, e.g. DMG selection, consensus sequence creation and primer design.
(XLSX)
