# Supplementary material for: The Identification of Novel Diagnostic Marker Genes for the Detection of Beer Spoiling Pediococcus damnosus Strains Using the BlAst Diagnostic Gene findEr
Source: PLoS One. 2016 Mar 30;11(3):e0152747. doi: 10.1371/journal.pone.0152747 (PMC4814128; doi:10.1371/journal.pone.0152747)
Supplement: S1 Table — In addition to beer spoilage potential category (spoilage-groups: strong beer spoilage potential (SB)—growth in pilsner (29 IBU). middle potential (MB)—growth in lager beer (18 IBU). weak potential (WB)—growth in wheat beer (14 IBU). no potential (NB)—no growth in test beers). OD590 and pH after 60 days of incubation are listed. (DOCX) [file pone.0152747.s001.docx]

**S1 Table.** Results of beer spoilage test. In addition to beer spoilage potential category (spoilage-groups: strong beer spoilage potential (SB) - growth in pilsner (29 IBU). middle potential (MB) - growth in lager beer (18 IBU). weak potential (WB) - growth in wheat beer (14 IBU). no potential (NB) - no growth in test beers). OD_590_ and pH after 60 days of incubation are listed.

| Strain | | Spoilage potential | Wheat beer | | Lager beer | | Pilsner beer | |
| --- | --- | --- | --- | --- | --- | --- | --- | --- |
|  |  | | OD_590_ | pH | OD_590_ | pH | OD_590_ | pH |
| TMW 2.4 | MB | | 0.06 +/- 0.00 | 4.18 +/- 0.00 | 0.07 +/- 0.02 | 3.97 +/- 0.00 | 0.00 +/- 0.00 | 4.39 +/- 0.00 |
| TMW 2.125 | NB | | 0.00 +/- 0.00 | 4.47 +/- 0.00 | 0.00 +/- 0.00 | 4.33 +/- 0.00 | 0.00 +/- 0.00 | 4.36 +/- 0.00 |
| TMW 2.1532 | NB | | 0.00 +/- 0.00 | 4.41 +/- 0.00 | 0.01 +/- 0.00 | 4.32 +/- 0.01 | 0.01 +/- 0.00 | 4.40 +/- 0.00 |
| TMW 2.1533 | SB | | 0.16 +/- 0.03 | 3.60 +/- 0.06 | 0.06 +/- 0.03 | 3.71 +/- 0.16 | 0.06 +/- 0.02 | 3.87 +/- 0.12 |
| TMW 2.1534 | NB | | 0.00 +/- 0.01 | 4.43 +/- 0.00 | 0.00 +/- 0.00 | 4.31 +/- 0.00 | 0.01 +/- 0.00 | 4.40 +/- 0.00 |
| TMW 2.1535 | SB | | 0.14 +/- 0.01 | 3.84 +/- 0.02 | 0.10 +/- 0.00 | 3.86 +/- 0.02 | 0.08 +/- 0.01 | 3.95 +/- 0.03 |
| TMW 2.1536 | NB | | 0.00 +/- 0.00 | 4.43 +/- 0.00 | 0.00 +/- 0.00 | 4.31 +/- 0.00 | 0.01 +/- 0.00 | 4.40 +/- 0.00 |
| TMW 2.1546 | NB | | 0.00 +/- 0.00 | 4.50 +/- 0.00 | 0.00 +/- 0.00 | 4.49 +/- 0.01 | 0.00 +/- 0.00 | 4.41 +/- 0.00 |
| TMW 2.1547 | NB | | 0.01 +/- 0.00 | 4.50 +/- 0.00 | 0.00 +/- 0.00 | 4.48 +/- 0.01 | 0.00 +/- 0.00 | 4.40 +/- 0.00 |
| TMW 2.1548 | MB | | 0.07 +/- 0.02 | 4.20 +/- 0.07 | 0.04 +/- 0.00 | 4.21 +/- 0.01 | 0.00 +/- 0.00 | 4.40 +/- 0.02 |
| TMW 2.1549 | NB | | 0.02 +/- 0.03 | 4.40 +/- 0.15 | 0.00 +/- 0.00 | 4.49 +/- 0.01 | 0.00 +/- 0.00 | 4.41 +/- 0.01 |
| TMW 2.1635 | NB | | 0.00 +/- 0.00 | 4.47 +/- 0.00 | 0.00 +/- 0.00 | 4.32 +/- 0.01 | 0.00 +/- 0.00 | 4.36 +/- 0.00 |
| TMW 2.1636 | SB | | 0.06 +/- 0.01 | 4.19 +/- 0.01 | 0.04 +/- 0.00 | 4.08 +/- 0.00 | 0.04 +/- 0.01 | 4.14 +/- 0.00 |
| TMW 2.1637 | SB | | 0.06 +/- 0.00 | 4.24 +/- 0.02 | 0.05 +/- 0.01 | 4.13 +/- 0.01 | 0.04 +/- 0.01 | 4.19 +/- 0.00 |
| TMW 2.1638 | NB | | 0.00 +/- 0.00 | 4.48 +/- 0.00 | 0.00 +/- 0.00 | 4.33 +/- 0.00 | 0.00 +/- 0.00 | 4.32 +/- 0.06 |
| TMW 2.1639 | NB | | 0.00 +/- 0.00 | 4.47 +/- 0.01 | 0.00 +/- 0.00 | 4.33 +/- 0.00 | 0.00 +/- 0.00 | 4.36 +/- 0.00 |
| TMW 2.1640 | NB | | 0.01 +/- 0.00 | 4.47 +/- 0.01 | 0.00 +/- 0.00 | 4.33 +/- 0.00 | 0.00 +/- 0.00 | 4.36 +/- 0.00 |
| TMW 2.1641 | SB | | 0.05 +/- 0.01 | 4.21 +/- 0.02 | 0.02 +/- 0.00 | 4.13 +/- 0.02 | 0.03 +/- 0.00 | 4.18 +/- 0.02 |
| TMW 2.1642 | NB | | 0.00 +/- 0.00 | 4.48 +/- 0.01 | 0.00 +/- 0.00 | 4.34 +/- 0.01 | 0.00 +/- 0.00 | 4.36 +/- 0.01 |
| TMW 2.1643 | NB | | 0.00 +/- 0.00 | 4.47 +/- 0.00 | 0.00 +/- 0.00 | 4.31 +/- 0.01 | 0.00 +/- 0.00 | 4.39 +/- 0.03 |
| non-inoculated beer |  | | 0 | 4.45 +/- 0.04 | 0 | 4.37 +/- 0.08 | 0 | 4.37 +/- 0.04 |
