# Supplementary material for: The Identification of Novel Diagnostic Marker Genes for the Detection of Beer Spoiling Pediococcus damnosus Strains Using the BlAst Diagnostic Gene findEr
Source: PLoS One. 2016 Mar 30;11(3):e0152747. doi: 10.1371/journal.pone.0152747 (PMC4814128; doi:10.1371/journal.pone.0152747)
Supplement: S2 Table — Genomes result from single molecule real time sequencing (Pacific Biosciences) and consequent hierarchical genome assembly process. Resulting contigs were manually curated and circularized. Annotation was performed using Rapid Annotations using Subsystems Technology (RAST) in default settings and RAST2BADGE. All biosamples are part of the bioproject PRJNA290141. Annotated features contain protein encoding genes as well as RNA features. TMW = Technische Mikrobiologie Weihenstephan. (DOCX) [file pone.0152747.s002.docx]

**S2 Table.** Genome metrics for sequenced strains. Genomes result from single molecule real time sequencing (Pacific Biosciences) and consequent hierarchical genome assembly process. Resulting contigs were manually curated and circularized. Annotation was performed using Rapid Annotations using Subsystems Technology (RAST) in default settings and RAST2BADGE. All biosamples are part of the bioproject PRJNA290141. Annotated features contain protein encoding genes as well as RNA features. TMW = Technische Mikrobiologie Weihenstephan

| Strain | Biosample | NCBI accession number(s) | Coverage | Genome size (Mbp) | Contigs | GC content (%) | Annotated features (RAST) |
| --- | --- | --- | --- | --- | --- | --- | --- |
| TMW 2.1532 | SAMN03876481 | CP012269 - CP012274 | 144 | 2.2 | 6 | 38.3 | 2134 |
| TMW 2.1533 | SAMN03876482 | CP012275 - CP012282 | 92 | 2.4 | 8 | 38.4 | 2333 |
| TMW 2.1534 | SAMN03876483 | CP012283 - CP012287 | 138 | 2.3 | 5 | 38.3 | 2236 |
| TMW 2.1535 | SAMN03876484 | CP012288 - CP012293 | 142 | 2.5 | 6 | 38.6 | 2404 |
| TMW 2.1536 | SAMN03876485 | CP012294 - CP012296 | 137 | 2.2 | 3 | 38.3 | 2091 |
