# Supplementary material for: The Identification of Novel Diagnostic Marker Genes for the Detection of Beer Spoiling Pediococcus damnosus Strains Using the BlAst Diagnostic Gene findEr
Source: PLoS One. 2016 Mar 30;11(3):e0152747. doi: 10.1371/journal.pone.0152747 (PMC4814128; doi:10.1371/journal.pone.0152747)
Supplement: S3 Table — Changing BADGE settings can be easily done using any text editor. Open BADGE.sh with your text editor and change settings as desired. Save your changes before you start a new BADGE run. We recommend to save a copy of BADGE.sh somewhere else. If your version does not work anymore (e.g. due to a typo), just replace it by the original BADGE.sh script. (DOCX) [file pone.0152747.s003.docx]

**S3 Table.** BADGE settings with input range, default value und description. Changing BADGE settings can be easily done using any text editor. Open *BADGE.sh* with your text editor and change settings as desired. Save your changes before you start a new BADGE run. We recommend to save a copy of *BADGE.sh* somewhere else. If your version does not work anymore, just replace it by the original *BADGE.sh* script.

| Setting name in BADGE | type | options | default value | description and notes |
| --- | --- | --- | --- | --- |
| clean_up | boolean | true/false | true | if switched on (true), only the most important output will be kept – all other files are deleted |
| min_DMG_occurrence | real | 0 - 1 | 1 | minimum occurrence of a DMG in group A to be reported, a value of 0.5 would mean, that the DMGs to identify only have to be in 50% of all members of group A in order to be reported |
| special_character | boolean | true/false | true | replaces incompatible special characters with ‘_’ |
| num_blast_proc | real | > 0 | 4 | number of parallel blast processes to start |
| megablast_perc_identity_cut | real | 0 - 100 | 95 | % identity threshold value – minimum identity of hit to be kept |
| megablast_e_value | real | > 0 | 1×10^-15^ | e-value threshold – maximum value of blast hit to be kept |
| megablast_within_group_qscov | real | 0 - 1 | 0.95 | Query / subject coverage (length of query divided by length of subject and vice versa), threshold within a group – minimum value of blast hit to be kept (relevant for core determination – Step 3) |
| megablast_between_group_qscov | real | 0 - 1 | 0.50 | Query / subject coverage threshold between groups – minimum value of blast hit to be kept (relevant for exclusion of ORFs – Step 1 and Step 2) |
| dc_mode | boolean | true/false | false | if switched on (true), basic DMG identification is done using dc-megablast instead of megablast, more sensitive but slower |
| dc_filter | boolean | true/false | true | if switched on (true), DC-megablast filter will be applied to data – potential DMGs with a long (> 50 % qscov) but low identity (default between 70 – 90 % identity) hit in other group will be discarded |
| dc_perc_identity_cut | real | 0 - 100 | 70 | c.f. megablast_perc_identity_cut |
| dc_blast_e_value | real | > 0 | 10 | c.f. megablast_e_value |
| dc_between_group_qscov | real | 0 - 1 | 0.50 | c.f. megablast_between_group_qscov |
| blastn_filter | boolean | true/false | true | if switched on (true), blastn filter will be applied to data – potential DMGs with a short (> 25 % qscov) but high identity (> 95 % identity) hit in other group will be discarded |
| blastn_perc_identity_cut | real | 0 - 100 | 95 | c.f. megablast_perc_identity_cut |
| blastn_e_value | real | > 0 | 10 | c.f. megablast_e_value |
| blastn_between_group_qscov | real | 0 - 1 | 0.25 | c.f. megablast_between_group_qscov |
| protein_level | boolean | true/false | false | if switched on (true), ORFs will be translated and used to perform BADGE (Steps 1 and 3) using blastp – NOTE: if Protein-Level is active dc-megablast filter and blastn filter are disabled automatically |
| blastp_perc_identity_cut | real | 0 - 100 | 50 | c.f. megablast_perc_identity_cut |
| blastp_e_value | real | > 0 | 10 | c.f. megablast_e_value |
| blastp_within_group_qscov | real | 0 - 1 | 0.50 | c.f. megablast_within_group_qscov |
| blastp_between_group_qscov | real | 0 - 1 | 0.50 | c.f. megablast_between_group_qscov |
| fastatranslate_geneticcode | real | 1 - 25 | 11 | genetic code to be used for translation – 11 corresponds to bacterial, archaeal and plant plastid code |
| fastatranslate_frame | real | 1 - 3 | 1 | reading frame to be translated |
| protein_level_clean_up | boolean | true/false | true | if switched on (true), translated orf files will be removed after BADGE is done |
| identify_overlapping | boolean | true/false | false | if switched on (true), overlapping DMGs are labelled correspondingly |
| mut_level_nt | boolean | true/false | false | if switched on (true) BADGE will look for DMGs with ANY (even single nucleotide changes) differences to the opposite group |
| mut_level_aa | boolean | true/false | false | if switched on (true) BADGE will look for DMGs with ANY (even single amino acid changes) differences to the opposite group in protein_level mode |
