# Supplementary material for: The Identification of Novel Diagnostic Marker Genes for the Detection of Beer Spoiling Pediococcus damnosus Strains Using the BlAst Diagnostic Gene findEr
Source: PLoS One. 2016 Mar 30;11(3):e0152747. doi: 10.1371/journal.pone.0152747 (PMC4814128; doi:10.1371/journal.pone.0152747)
Supplement: S7 Table — Primer sequences were created from consensus sequences of all available open reading frames of a given DMG. PCR was performed at 50°C for all marker genes. horA, horC and hitA are DMGs which were not identified within this study. (DOCX) [file pone.0152747.s007.docx]

**S7 Table.** DMG specific primer pairs. Primer sequences were created from consensus sequences of all available open reading frames of a given DMG. PCR was performed at 50 °C for all marker genes. *horA*, *horC* and *hitA* are DMGs which were not identified within this study.

| DMG / primer designation | forward sequence | reverse sequence | product size (bp) |
| --- | --- | --- | --- |
| parA_f / parA_r | ATCGTGCTATGTTCGCACTC | CAAGGCCATCAGCACTTATCTC | 168 |
| tetR_f / tetR_r | TACACCGCGGCAATTGAAG | GGGTTCGATAATCGCGTTCAG | 147 |
| tnpA_f / tnpA_r | GGCAAACGGGTTGAATCTG | AGTGCTCGGTTCCATAGTC | 148 |
| fabZ:f / fabZ_r | ATTGAGGCAATGGCTCAGAC | CGATCCGTGACCTAATCCAATG | 186 |
| icaA_f / icaA_r | GCGGTTTAAGCGGGATTAC | CGTCACAATGCCGTCATTC | 172 |
| nplA_f / nplA_r | TCTTGCAGCCTGGTACAC | CGTCGCAATGCCACTAAC | 154 |
| galM_f / galM_r | ATAGCGCAAAGCAGCATC | CAAATCCGTCGTGGTGAATC | 174 |
| hypA_f / hypA_r | TATCTCCGTCCTGGGATTTG | GGGACCGTTTGGTAGCTTAG | 193 |
| tnpB_f / tnpB_r | TAAGGCATCACGGCAAGTC | CCTAACACGGTATCACCTTCC | 145 |
| npxA_f / npxA_r | TCCAGCAGGTAAGCCAATG | CGCAGTAGCGAAGTGATAGTC | 160 |
| horA_f / horA_r | AATCTTAACCCTGCCGGTGG | TGGATTCGAGTGGTTGAGCC | 636 |
| horC_f / horC_r | TACACAGAAACCCGTTCACC | CTGTGCGCTAATTCGTGATG | 133 |
| hitA_f / hitA_r | TTGCAATCAATGGCTGCTCG | TGCGGTCCCGCTAAGAATAC | 338 |
